# The Solitary Fibrous Tumor, the Chameleon of the Head and Neck Region—Clinical, Epidemiological, and Histopathological Aspects

**DOI:** 10.3390/diagnostics15212721

**Published:** 2025-10-27

**Authors:** Marina Rakitovan, Adrian Nicoara, Raluca Maria Closca, Raluca Amalia Ceausu, Cristina Stefania Dumitru, Alina Cristina Barb, Dorin Novacescu, Antonia Armega Anghelescu, Alexandru Cristian Cindrea, Flavia Zara

**Affiliations:** 1 Department of Microscopic Morphology, University of Medicine and Pharmacy “Victor Babes”, 300041 Timisoara, Romania; 2 Oro-Maxillo-Facial Surgery Clinic, Emergency City Hospital, 300062 Timisoara, Romania; 3 Angiogenesis Research Center, University of Medicine and Pharmacy “Victor Babes”, 300041 Timisoara, Romania; 4 Discipline of Dentoalveolar Surgery, University of Medicine and Pharmacy “Victor Babes”, 300041 Timisoara, Romania; 5 Department of Pathology, Emergency City Hospital, 300254 Timisoara, Romania; 6 Doctoral School, Faculty of General Medicine, University of Medicine and Pharmacy “Victor Babes”, 300041 Timisoara, Romania; 7 Emergency Clinical Municipal Hospital, 300254 Timisoara, Romania

**Keywords:** head and neck pathology, maxillofacial pathology, oral pathology, solitary fibrous tumor, mesenchymal tumors, immunohistochemical reaction

## Abstract

**Background/Objectives:** The solitary fibrous tumor is an uncommon benign mesenchymal neoplasm with relatively indolent and rarely metastasizing behavior. This retrospective study includes 26 cases of head and neck solitary fibrous tumors diagnosed between 2017 and 2024. **Methods:** The morphological examination with Hematoxylin–Eosin staining was completed via immunohistochemical reactions with specific antibodies. **Results:** The Ki-67 proliferation index had a median of 11.2%, with an interquartile range of 5% to 15%. CD20-positive B-cells had a score of 0 in 50% of cases (*n* = 26), while CD3 and CD5 T-cells had a score of 3 in 81% of cases (*n* = 21). CD4-positive T-cells had a majority score of 1 (81%, *n* = 21). CD8-positive T-cells had a broader distribution: 65% (*n* = 17) of cases presented a score of 1, 27% (*n* = 7) a score of 2, and 8% (*n* = 8) a score of 0. Antigen-presenting dendritic cells and mast cells presented a majority score of 0 in the entire cohort, being undetectable in 85% (*n* = 22) and 88% (*n* = 23) of cases, respectively. CD20-positive B-lymphocytes demonstrated moderately strong correlations with the Ki-67 cell proliferation index (r = 0.77). The time to recurrence was most strongly associated with the Ki-67 mitotic index (r = 0.81), CD4-positive (r = 0.85), and CD5-positive T-lymphocytes (r = 0.55), and CD20-positive B-lymphocyte expression (r = 0.68). **Conclusions:** This research illustrates our experience with head and neck solitary fibrous tumors, the surgical decisions, and the morphological and immunohistochemical features, while reviewing the cases published in English in the specialized literature.

## 1. Introduction

The solitary fibrous tumor (SFT) represents a generally benign, uncommon neoplasm that belongs to the range of mesenchymal tumors [1,2,3]. By the WHO Classification of Soft Tissue and Bone Tumors, the solitary fibrous tumor is classified as a fibroblastic neoplasm with relatively indolent and rarely metastasizing behavior [1,4,5,6]. Solitary fibrous tumors were considered distinct from hemangiopericytomas after Klemperer et al. first described them in 1931 as a separate entity of mesothelial origin arising in the pleura [7,8,9,10]. The knowledge about the two entities, solitary fibrous tumor and hemangiopericytoma, has developed since their first representation [11]. Specifically by finding the intrachromosomal inversion succeeding from NAB2-STAT6 gene fusion, tumors previously known as hemangiopericytomas became acknowledged as a cellular type of the classic solitary fibrous tumor [7].

Throughout time, numerous terminologies were used for solitary fibrous tumors, namely localized mesothelioma, benign mesothelioma, subpleural fibroma, submesothelial fibroma, subserosal fibroma, solitary fibrous mesothelioma, and localized fibrous tumor, which highlights its ever-evolving pathogenesis [4,8,9,12,13]. The occurrence mainly affects middle-aged adults, without any gender predilection [1,6,11,12,14,15,16]. The “parent cell” from which the solitary fibrous tumor originates has not been clearly identified [17]. Originally, it was noted to appear specifically in the pleura and lungs; however, at present, it can be found at any site [1,6]. Of the sites documented, 50–70% arise extrathoracic, on sites such as proximal extremities, vulva, abdomen, retroperitoneum, trunk, thyroid gland, and meninges [2,6,14]. In 1991, 60 years after Klemperer et al., Witkin et al. were the first to report cases of solitary fibrous tumors in the head and neck (H&N) region [8,18]. The head and neck area is an uncommon site, accounting for 6–18% of all solitary fibrous tumors and approximately a quarter of the extrathoracic ones [6,12].

Solitary fibrous tumors comprise pleomorphic spindle cells and collagen fibers, with a disorganized, diffused structure, or, at most, disposed in short fascicles [1,12]. The clinical behavior of this tumor is quite unpredictable (i.e., it can impersonate both benign and malignant soft tissue tumors), making the process of diagnosis rather challenging due to its histological diversity, especially at unusual sites, such as the head and neck area [1,4,6,11,19].

## 2. Materials and Methods

### 2.1. Inclusion Criteria of the Patients

The data found in this manuscript represent a retrospective study with a time span of 7 years, between 2017 and 2024. It included 37 cases of head and neck solitary fibrous tumors diagnosed in the Pathology Department of Emergency City Hospital from Timisoara, Romania. The biopsies were performed in the Oro-Maxillo-Facial Surgery Clinic and Ear, Nose, and Throat Clinic. The registries of the Pathology Department were used in order to identify the cases.

**Figure 1:**
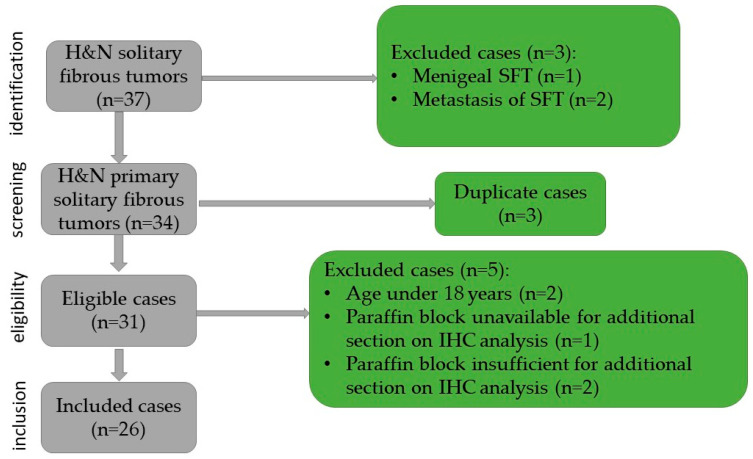
Flowchart of the case selection.

The inclusion criteria in the study were patients age over 18 years, head and neck tumor site, histopathological examination, and confirmation of solitary fibrous tumor, availability of a paraffin block sufficient for immunohistochemical (IHC) staining, and the presence of consent for publication. In this study, 26 cases were included, based on the exclusion criteria as follows: meningeal solitary fibrous tumors, metastasis of solitary fibrous tumors, duplicate cases, paraffin block unavailable or insufficient for additional sections, immunohistochemical analysis, and age under 18 years old (Figure 1). For each case, the age, gender, symptomatology, the site and size of the lesion, as well as the surgical approach and recurrence were identified.

### 2.2. Ethical Considerations

In accordance with the ethical standards imposed by the Declaration of Helsinki and Romanian legislation, the approval of the hospital ethical commission was secured. The informed patient consent for the use of histologically processed biological samples for diagnostic and scientific purposes was certified.

### 2.3. Laboratory Process

The harvested tissue was prepared by standard histological procedure, with the obtainment of the paraffin blocks, and subsequent histopathological examination was performed. Tree micrometer thick serial sections were cut on a Leica RM2245 semi-automated rotary microtome (Leica Biosystems, Nussloch, Germany), displayed on SuperFrost™ microscope (St. Louis, MO, USA) slides, for all the cases. The initial stain used for morphological examination was Hematoxylin–Eosin. The histopathological aspects were noted for each tumor as follows: architectural pattern of the tumor growth, dominant constituent cell, mitotic index counted per 10 high-power fields, cellular atypia, areas of necrosis, peritumoral cellular immune response, and assessment of the resection margins.

The pathological diagnosis was completed using an immunohistochemical profile with specific antibodies such as: anti-STAT6, anti-CD34, anti-EMA, anti-Bcl2, anti-SMA, anti-S100 protein, anti-desmin, anti-CD99, anti-Ki-67 index, anti-CD20, anti-CD3, anti-CD4, anti-CD5, anti-CD8, anti-CD1a, and anti-CD117. All of the technical data related to the type of antibodies used, as well as their clone and dilution, are presented in Table 1.

All of the antibodies and the reagents used for immunohistochemistry came from Novocastra™ (Leica Biosystems, Newcastle, UK), and the immunohistochemical staining was carried out with the Leica Bond-Max automatic instrument (Leica Biosystems Melbourne Pty Ltd, Mount Waverley, Australia), following the manufacturer’s recommended protocol.

Sections for immunohistochemistry study were performed sequentially, and each three-micrometer-thick tumor slice was stained with a single antibody. The evaluation was performed individually and manually by two pathologists with experience in head and neck pathology, using a Leica DM750 microscope. The images were secured using the Leica DM Share system. The results of the immunohistochemical reaction were recorded, specifying the intensity (weak, moderate, or high) and the pattern of the reaction (diffuse or focal), as well as the percentage of positive cells compared to the total tumor cells present in the field. The evaluation of the tumor immune microenvironment was performed using a scoring system, ranging from 0 to 3, as follows: score 0: ≤9, score 1: 10–49, score 2: 50–99, and score 3: >100 positive cells per 10 high-power fields. The risk of tumor recurrence was assessed using the risk stratification systems proposed by Demicco, Sugita, G-score, Huang, Salas, and Thompson [1,20,21,22,23,24]. Interindividual variations were corrected by subsequent discussion and agreement.

### 2.4. Objectives

The first objective was to present the main clinical, epidemiological, and morphological data of solitary fibrous tumors of the head and neck region, along with their classification, according to existing risk stratification systems. The second objective was to identify and quantify the cells of the tumor immune microenvironment. Finally, the third objective was to identify any statistical correlations between the immune cells of the tumor microenvironment and different parameters.

### 2.5. Data Analysis

Data analysis has been conducted using R 4.3.2 packages corrplot, dplyr, pROC, readxl, and tidyverse.

Descriptive statistics were computed for all variables. Continuous variables are summarized using the median and interquartile range (IQR), reflecting the central tendency and dispersion of the data. Categorical variables are presented as absolute counts and corresponding percentages. Associations between variables were evaluated using the Spearman rank correlation coefficient. To assess the predictive performance of each scoring system for tumor recurrence, we determined the area under the receiver operating characteristic curve (AUC), sensitivity, and specificity. The AUC was used to quantify the overall discriminative ability of each score, while sensitivity and specificity provided insights into their ability to correctly classify patients with and without recurrence, respectively. Where appropriate, 95% confidence intervals (CIs) were reported for these estimates to convey the precision of the results.

## 3. Results

### 3.1. Clinical and Epidemiological Aspects

This study includes 26 cases of solitary fibrous tumor of the head and neck region. We would like to mention that the term “case” refers to the primary tumor itself. The median age value at diagnosis was 55, with a male predominance. The peak incidence for males was in the 3rd and the 5th decade (*n* = 4, 15%), and for females in the 4th decade (*n* = 4, 15%). Additional clinical, epidemiological, and data revealing the treatment and outcome of the tumors, as well as recurrence data, are summarized in Table 2, Supplemental Tables S1 and S2.

Most tumors were located in the auricular and periauricular area (*n* = 7, 27%), the nasal region (*n* = 6, 23%), the orbital and periorbital region (*n* = 5, 19%), and the labial region (*n* = 2, 8%). The rest of the cases were located as follows: nasopharyngeal, oropharyngeal, latero-cervical, maxillary, and jugal region, one case for each site. The median size of the lesion was 1.3 cm. The clinical presentation varied among patients according to the lesion’s site and size. Nonspecific symptoms were marked, such as unilateral hearing loss, visual acuity, discomfort in mastication, odynophagia, and even dysphagia, dysphonia, rhinorrhea, bilateral nasal obstruction, dyspnea, and consequential oral breathing (Supplemental Table S1).

Due to the small dimensions of the lesions and their sites, for most cases, imaging investigations were not performed. Most of the masses were clinically or endoscopically accessible.

### 3.2. Surgical Approach

All the patients mentioned in this manuscript underwent surgical intervention, either excisional or incisional biopsy of the tumor. Due to the localization of the tumor, the extraoral cutaneous surgical approach was performed in 15 cases, the endonasal approach was elected for five cases, while six cases had an intraoral surgical approach. General anesthesia with orotracheal intubation was elected for nine cases, two cases being treated under intravenous anesthesia, while for 15 cases, a local anesthesia was performed. A complete surgical excision was performed for 21 cases. Intraoperative, no invasiveness of the lesion in the surrounding tissues was observed, and no macroscopic residual tissue remained postoperatively. For the remaining five cases, an incisional biopsy was decided upon (Supplemental Table S2).

### 3.3. Morphological Features

**Figure 2:**
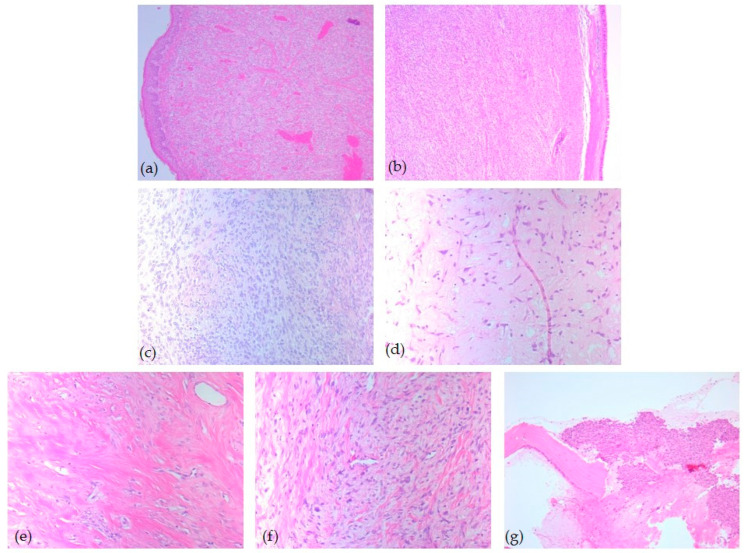
Head and neck SFT, microscopic aspects—HE-stained slides: (**a**) Classic subtype with spindle cells, ob. 5×; (**b**) Cellular variant with spindle cells, ob. 5×; (**c**) Cellular variant with epithelioid and spindle cells ob. 20×; (**d**) Mixoid variant, ob. 20×; (**e**) Paucicellular sclerotic collagen subtype, ob. 20×; (**f**) Classic subtype with nuclear atypia, ob. 20×; (**g**) Mixoid subtype with osseous invasion ob. 10×.

Microscopic examination revealed that most tumors (*n* = 14, 54%) presented the classic variant of solitary fibrous tumor, with a patternless growth or a storiform pattern, being composed of spindle or epithelioid cells, disposed in short wavy fascicles or haphazardly bundles. The tumor stroma showed a dilated, branched, hyalinized vasculature (hemangiopericytoma-like) and hyalinized collagen bands. The classic variant of solitary fibrous tumor was paucicellular. In contrast, the cellular variant (*n* = 5, 19%) presented increased cellular density, with the constitution of short, overlapping bundles of tumor cells. Six tumors showed myxoid degeneration of the stromal tissue and a low/moderate cell population. Also, one case (4%) has a particular histopathological variant characterized by very low cellularity and markedly sclerosing stroma (Figure 2).

Minimal cellular atypia, defined by the presence of less than 1% atypical cells with slightly enlarged nuclei, was identified in most patients (*n* = 17, 65%), and moderate cellular atypia with up to 10% enlarged and hyperchromic nuclei, in four patients (15%). Five patients (19%) presented marked cellular atypia defined by nuclear overlap, high pleomorphism with hyperchromatic nuclei, and foci of bizarre cells.

Half of the presented tumors (*n* = 13) showed low mitotic activity, with 1–2 mitoses per 10 fields at 40× objective. The rest of the tumors had intermediate mitotic index, with 3–4 mitoses (*n* = 8), respectively. A total of 5 cases had very high mitotic activity, with 5, 7, or even 8 mitoses per field at 40× objective. Areas of tumor necrosis were reported as absent or minimally focal (<10% of the examined areas). Although most tumors did not show necrosis, 3 cases (12%) had focal areas of tumor necrosis (Table 3 and Supplemental Table S3).

### 3.4. Immunohistochemical Features

The immunohistochemical study revealed that most tumors showed diffuse and intense reaction for specific diagnostic markers of solitary fibrous tumors, namely STAT 6 (*n* = 26, 100%), CD34 (*n* = 25, 96%), Bcl2 (*n* = 26, 100%) and CD99 (*n* = 21, 81%) and were negative for S100 protein and desmin in 100% of cases. The Ki-67 proliferation index had a mean of 11.2%, with an interquartile range (IQR) of 5% to 15%, consistent with generally low-to-intermediate proliferative activity across the cohort (Table 4).

**Figure 3:**
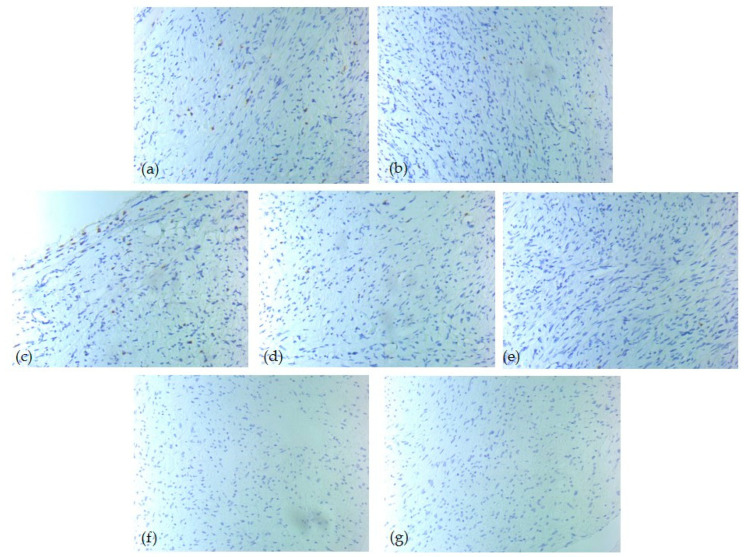
IHC tumor immune microenvironment profile—one low-risk case of head and neck SFT, ob. 20xs: (**a**) CD3 positive T-lymphocytes, score 2; (**b**) CD4 positive T-lymphocytes, score 1; (**c**) CD5 positive T-lymphocytes, score 2; (**d**) CD8 positive T-lymphocytes, score 1; (**e**) CD20 positive B-lymphocytes, score 0; (**f**) CD1a positive dendritic cells, score 0; (**g**) CD117 positive mast cells, score 0.

**Figure 4:**
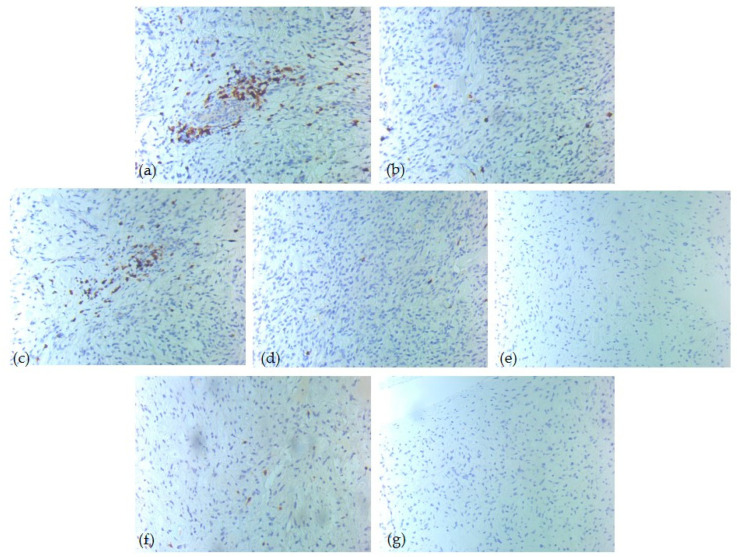
IHC tumor immune microenvironment profile—one intermediate-risk case of head and neck SFT, ob. 20xs: (**a**) CD3 positive T-lymphocytes, score 3; (**b**) CD4 positive T-lymphocytes, score 1; (**c**) CD5 positive T-lymphocytes, score 3; (**d**) CD8 positive T-lymphocytes, score 1; (**e**) CD20 positive B-lymphocytes, score 0; (**f**) CD1a positive dendritic cells, score 1; (**g**) CD117 positive mast cells, score 0.

**Figure 5:**
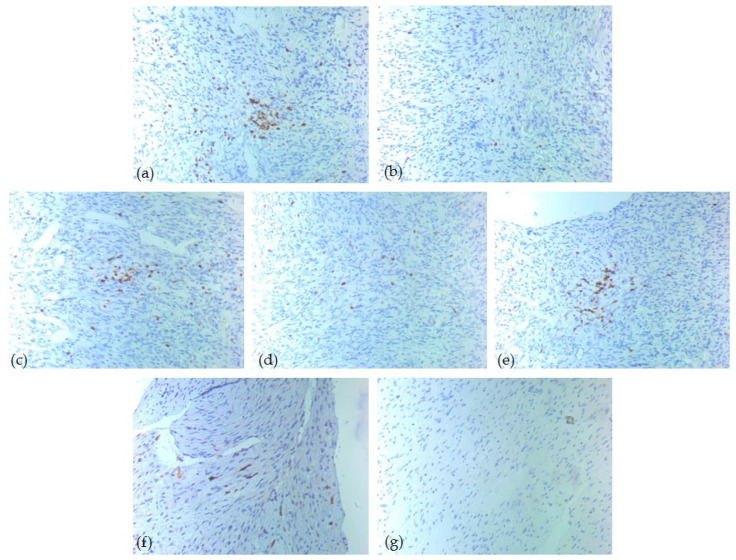
IHC tumor immune microenvironment profile—one high-risk case of head and neck SFT, ob. 20xs: (**a**) CD3 positive T-lymphocytes, score 3; (**b**) CD4 positive T-lymphocytes, score 1; (**c**) CD5 positive T-lymphocytes, score 3; (**d**) CD8 positive T-lymphocytes, score 1; (**e**) CD20 positive B-lymphocytes, score 3; (**f**) CD1a positive dendritic cells, score 1; (**g**) CD117 positive mast cells, score 0.

Immunohistochemical analysis revealed heterogeneous expression patterns among the tumor immune microenvironment cells (Table 5). CD20-positive B-lymphocytes had a score of 0 in 50% of our cases, while 27% of cases had a score of 3. In contrast, CD3 and CD5 showed a score of 3 in 81% of cases, suggesting strong T-cell lineage activity in those samples. CD4-positive T-lymphocytes presented mostly a score of 1 (81%), with a score of 0 in only 4%. CD8 showed a broader distribution: 65% of cases noted score 1, 27% score 2, while 8% score 0. CD1a and CD117 presented mostly score 0 across the cohort, being undetectable in 85% and 88% of cases, respectively (Figure 3, Figure 4 and Figure 5).

**Figure 6:**
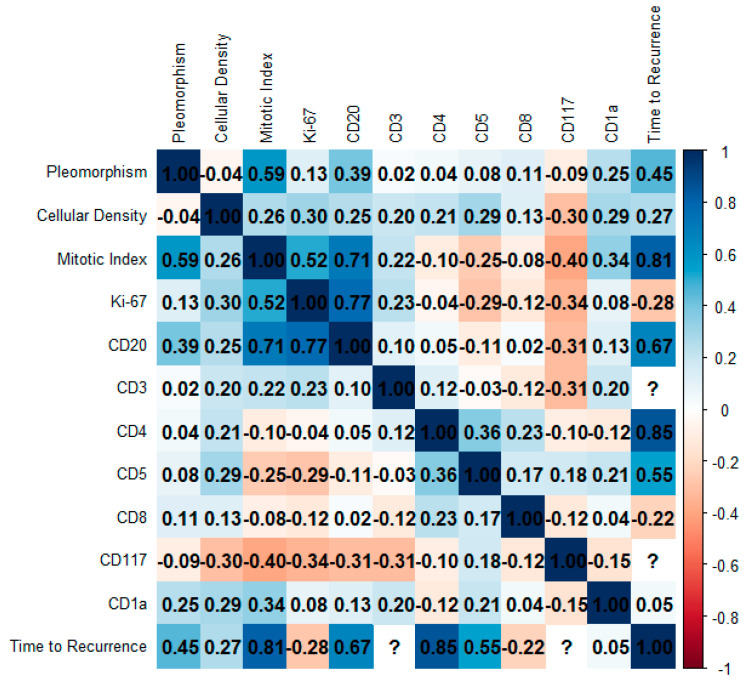
Spearman correlation matrix representing the relationships among histopathological and immunohistochemical parameters and time to recurrence in the study cohort. Variables include morphological features (Pleomorphism, Cellular Density, Mitotic Index), proliferative marker (Ki-67), and immune markers (CD20, CD3, CD4, CD5, CD8, CD117, CD1a). Correlation coefficients range from −1 to 1 and are visualized through a diverging color scale (blue for positive, red for negative correlations), with the magnitude indicated numerically within each cell. Notably, strong positive correlations were observed between Mitotic Index and Time to Recurrence (r = 0.81) and between CD4 and Time to Recurrence (r = 0.85), suggesting potential prognostic relevance. Cells marked with “?” indicate missing or non-computable values.

Figure 6 presents a Spearman correlation matrix assessing the relationships between key histopathological variables, tumor immune microenvironment cells, and clinical outcome, specifically time to recurrence, in the analyzed cohort. A strong positive correlation was identified between mitotic index and Ki-67 index expression (r = 0.52), reinforcing the proliferative relevance of the Ki-67 labeling index. CD20-positive B-lymphocytes demonstrated consistent and moderately strong correlations with both mitotic index (r = 0.71) and Ki-67 proliferation index (r = 0.77), suggesting an association between B-cell marker expression and proliferative tumor behavior. Time to recurrence was most strongly associated with mitotic index (r = 0.81), CD4-positive T-cells (r = 0.85), and CD20-positive B-cells expression (r = 0.68), indicating that higher proliferative activity and the presence of specific immune cell markers may be linked to delayed recurrence. CD5-positive T-cells also showed a moderate correlation with time to recurrence (r = 0.55), further implicating T-cell lineage markers in disease progression. Several variables, including CD3-positive T-cells and CD117-positive mast cells, could not be evaluated due to insufficient data or lack of computational feasibility; these are indicated by question marks within the matrix. The use of a diverging color scale allows for visual differentiation between positive and negative correlations, with the numerical values facilitating interpretation of magnitude. Overall, the observed correlations underscore a potential prognostic role for mitotic activity and immune marker expression in predicting clinical outcomes.

### 3.5. Risk Stratification Scores

Risk stratification was performed using six previously established scoring systems: Demicco, Sugita, G-score, Huang, Salas, and Thompson (Table 6). Across all systems, most cases were classified as low risk, ranging from 58% (Sugita) to 96% (Salas). Intermediate-risk assignments varied more widely, with the Sugita score classifying 38% of cases in this category, compared to only 4% in the Salas system. High-risk designations were rare across all scoring models, with only a single case classified as high risk in the Sugita, Huang, and Thompson scores (each representing 4% of the cohort) and three cases (12%) in the Demicco system. The G-score and Salas score did not identify any high-risk patients.

**Figure 7:**
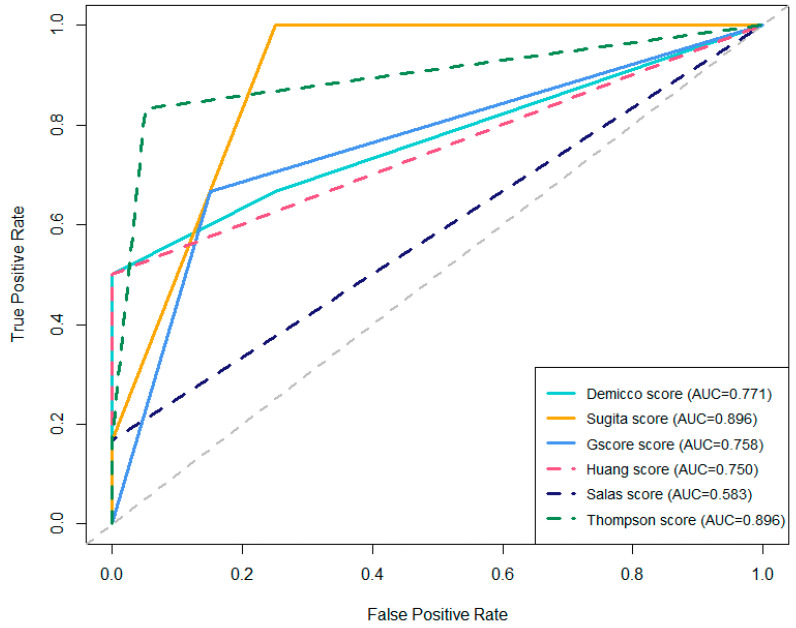
Comparative ROC Curves of prognostic scores for tumor recurrence.

As demonstrated in Table 7 and Figure 7, the comparative analysis of multiple prognostic scores for tumor recurrence reveals notable differences in predictive performance. The Sugita and Thompson scores achieved the highest AUC, both with values of 0.896, indicating excellent discriminatory ability. In particular, the Sugita score demonstrated the highest sensitivity (100%), although with a lower specificity (75%). The Thompson score provided a balanced profile, with high sensitivity (83.3%) and high specificity (95%). In contrast, the Demicco score, while showing perfect specificity (100%), had a lower sensitivity (50%) and a moderately high AUC of 0.771. Among the remaining scores, the G-score and Huang score achieved AUCs of 0.758 and 0.750, respectively, with moderate sensitivity and high specificity. The Salas score exhibited the lowest predictive performance (AUC = 0.583), with markedly low sensitivity (16.7%) despite perfect specificity. The ROC curves presented in Figure 7 visually summarize these findings, illustrating the trade-offs between sensitivity and specificity for each scoring system.

### 3.6. Outcome

Recurrence was noted in five cases, including patients who had prior surgery, those who developed it after completing chemotherapy or radiation therapy, and even despite complete surgical excision. The time of recurrence ranged between 3 and 168 months, with the average value of 45 months. A single case exhibited four recurrences at 14, 28, 34, and 42 months after the initial diagnosis of the primary tumor (Supplemental Table S2).

Positive surgical margins were not found in our cases, although at the histopathological exam, a local invasion was mentioned in three cases. Merely a few of the patients were sent for postoperative oncological consultation, of which four underwent adjuvant treatment. Specifically, three patients had radiotherapy treatment, and one patient had chemotherapy treatment. To the best of our knowledge, none of the patients developed distant metastasis or died of this tumoral disease, even though a loss of one patient was noted—a death caused by an aggravated general cardiorespiratory condition.

## 4. Discussion

Solitary fibrous tumor is an atypical benign neoplasm consisting of spindle cells and collagen fibers, disposed in a disorganized pattern [1,2,3]. As for the clinical features of this tumor, they are, to say the least, inconsistent [1].

The appearance of this tumor seems to have no gender predilection, yet Cox et al. mentioned that 44% of cases were male and 56% female [2,6,9,11,14,15,16,25]. In contrast, our findings suggested a male predominance.

The extraconal and intraconal spaces of the orbit, including the conjunctiva, sclera, eyelids, and lacrimal sac, as well as ethmoid and sphenoid sinuses, are frequently involved. The sinonasal tract (the nasal cavity or paranasal sinuses, alone or in combination), the ear and external auditory canal, the oral cavity including soft palate and palatine tonsils; furthermore larynx, epiglottis, parotid and submandibular gland, parapharyngeal spaces, as well as the neck area represent the most common head and neck sites for the solitary fibrous tumors [2,4,6,7,11,14,25,26,27,28]. Regarding the cases presented in this research, the auricular and periauricular sites were the most common sites, followed by the nasal, orbital/periorbital, and labial sites. Nasopharyngeal, oropharyngeal, cervical, maxillary, and jugal regions represent the rarest localization of solitary fibrous tumor of the head and neck region.

The symptomatology of the solitary fibrous tumor is nonspecific [9]. In some patients, these tumors can be present as an asymptomatic, indolent, progressively enlarging facial or cervical masses, while other patients can report symptoms represented by tumor mass effect such as swelling, unilateral nasal obstruction, epistaxis, ptosis, proptosis, diplopia or even changes in visual field, as well as vocal transformation, upper tract respiratory suffering or headache [6,8,9,15,29,30]. Constrictive symptoms may develop while the tumor is still of a small size, especially in the orbit area or the nasal cavity. A lower percentage of patients experience pain (11%), which is usually associated with larger tumors [6,8,15].

The visibility of the mass or the awareness of the local symptoms tends to encourage patients with head and neck tumors to an earlier presentation, a fact that can, in the long run, limit the extent of disease [8].

Certain radiographic findings could be suggestive of the diagnosis of solitary fibrous tumors [8]. Taking into consideration imaging investigations, solitary fibrous tumors are viewed as heterogeneous masses associated with a well-delimitated neoplastic process, yet local infiltration of adjacent osseous, nervous, and circulatory systems has been found in some cases (13%) [6,8,12,15,31]. Computed Tomography scans with contrast reveal variable degrees of heterogenous alterations in head and neck solitary fibrous tumors [6,8,29]. The imaging of erosive bone changes should raise concern for malignancy, yet the absence of this aspect does not exclude the possibility of a malignant type tumor [8,29]. Computed Tomography remains the imaging of choice for the evaluation of the solitary fibrous tumors [12]. The Magnetic Resonance Imaging and signal characteristics of the tumors compared to adjacent structures could also help in the process of diagnosis [8]. It is used in evaluating the delineation of the tumor and the local invasion into adjacent structures [12,32]. In our cases, Magnetic Resonance Imaging and Computed Tomography were performed for three cases each.

Macroscopically, solitary fibrous tumors are usually circumscribed, well-defined, lobulated, partially encapsulated tumors, presented by a white, gray, tan, or reddish-brown color [6,8,11,14]. The size of the tumor can vary from 0.4–18 cm, with the median size of 2.5–4 cm, depending on the localization of the tumor: in the oral cavity, being significantly smaller (2 cm) than in the sinonasal tract and neck area (5 cm) [4,6,11,14,15,26]. The consistency is mostly solid. These tumors are rather difficult to differentiate from other soft tissue tumors [2,6].

Proceeding with the microscopic part, originally, solitary fibrous tumor and hemangiopericytoma were noted as two separate entities [6]. Afterwards, it was found that those two should be classified on the other end of the scale, due to clinicopathological and histopathological overlap [6]. Finding of the NAB2-STAT6 gene merged these two entities, resulting in the acceptance of the term “solitary fibrous tumor” for all these tumors, while abandoning the terminology of hemangiopericytoma [6]. Therefore, hemangiopericytoma is now thought to be a cellular type within the spectrum of the solitary fibrous tumors [1].

Based on their prevailing component, four types of solitary fibrous tumors were distinguished: classic, cellular, myxoid, and fibrous or paucicellular sclerotic collagen type [14]. Microscopically, Hematoxylin–Eosin staining showed ovoid or spindle-shaped cells, displayed in patternless arrangements, with alternating hypocellular and hypercellular areas, some intervening medium-sized blood vessels characterized as “staghorn” vasculature, and abundant keloid-like collagen fibers, often with artifactual spaces present between the group cells and the collagen bundles [6,8,9,11,14,17]. Cellular tumors may present fascicular, storiform, herringbone, epithelioid, or sheet-like patterns [6,14]. The cells hold small, round or oval nuclei, vesicular chromatin, frequent nuclear pseudo inclusions, and discreet nucleoli [4,6,14,16,25]. Tumor cells may also present cytologic atypia and even epithelioid morphology, as well as some surrounding fibrosis and/or hyalinization [4,6,16,25].

Tariq et al. reported those traditional histological features of the solitary fibrous tumors in all of their cases, as well as some of the inconsistent microscopic aspects that contribute to the variety of solitary fibrous tumors, including: infiltrative growth patterns (26–49% of cases), myxoid degeneration (3–45%), micro- and macrocysts (7–43%), fusiform giant cells (7–10%), dedifferentiated areas (1%), lipomatous change (3–19%), giant-cell angiofibroma-like areas (17%), areas of coagulation necrosis (6%) and nodular hyaline deposits (with collagen rosettes or amianthoid fibers; 5%) [4,6,11,14,16].

Marti-Flich et al. found that CD34 presented strong positivity in most cases, while two of their 34 patients were CD34-negative [12]. The group showed local recurrence in five cases and a low risk of metastasis [12]. Similar findings were noted in our group of patients. Moreover, the finding of the intrachromosomal rearrangement on chromosome 12q13 confirms the nuclear expression of STAT6 protein [12].

Immunohistochemistry has a primary role in the microscopic diagnosis of solitary fibrous tumors [7]. Some studies showed that the recurrent NAB2-STAT6 gene fusion and STAT6 immunohistochemical expression are present in almost all cases, the last one being especially helpful in identifying CD34-negative solitary fibrous tumors [4,6,11,15,16,25,33]. Even though STAT6 expression may be present in other mesenchymal tumors, it regularly reveals nuclear positivity in solitary fibrous tumors [6,15,16]. CD34 expression has been found in 90–98% of cases [4,6,11,15,16]. CD34 and STAT6 are important immunohistochemical antibodies for establishing the diagnosis of solitary fibrous tumors, in addition to other immunohistochemical stains as well [4,6,11,15,16,25]. Cluster of differentiation group 34 is a single-pass type I transmembrane glycoprotein [12]. Its main function is a cell-to-cell adhesion factor; a factor that is expressed during the lymphohematopoietic or progenitor stage of development and may eventually mediate stem cell attachment to bone marrow, extracellular matrix, or stromal cells [12]. In the head and neck area, CD34-negative solitary fibrous tumors seem to appear more frequently, although showing a more aggressive behavior [12]. On the other hand, expression of STAT6 protein is considered the most specific marker for the diagnosis of solitary fibrous tumors [12]. Furthermore, it was observed that the focal or multifocal expression for Bcl2, CD99, S100, SMA, EMA, and pan cytokeratin is also present in 75–94%, 91–93%, 6–16%, 8%, 2–5%, and 4% of cases, following the previously mentioned order [6,11,15]. Head and neck solitary fibrous tumors are negative for desmin, NKX2.2, SOX10, CD31, ERG, synaptophysin, and chromogranin A [6,14]. Other studies show that solitary fibrous tumors express CD34 in 80–95% cases and CD99 in 70%, while infrequently expressing Bcl2, epithelial membrane antigen, and smooth muscle actin (20–35% of cases) [8]. A few isolated cases of focal and limited reactivity for S-100 have been reported [8]. The possibility of solitary fibrous tumors should be ruled out in cases that show positive results to testing of the NAB2-STAT6 gene fusion [6]. Diversity in the panel of markers used for the immunohistochemistry by each researcher further results in heterogeneity of the outcome [12]. Chung et al. reported that 100% were positive for STAT6, vimentin, and CD99. A total of 97.9% of tumors were positive for CD34, 90.9% for Bcl2, 33.3% for SMA, while only 19.5% for S-100 [7]. The cases presented in this article related to almost similar results to those noted in the literature. Specifically, all tumors were positive for STAT6, while only 81% for the CD99 marker. Regarding CD34 and Bcl2, our data were identical to those found in the literature. Furthermore, 96% were positive for CD34 and 100% were positive for Bcl2 protein, with the intensity reaction ranging from low to high.

The morphological spectrum of the head and neck solitary fibrous tumors expands due to cellular variability, as well as stromal changes [11,34]. Multiple variants and concomitantly, multiple differential diagnoses can be found [6,34].

Differential diagnosis of solitary fibrous tumors of the head and neck area includes smooth muscle tumors like leiomyoma, schwannoma, epithelioid hemangioendothelioma, juvenile angiofibroma, glomus tumor, paraganglioma, and giant-cell fibroblastoma [14,35,36].

The differential diagnosis of malignant solitary fibrous tumors was numerous: the anaplastic carcinoma, in cases involving the neck region and the thyroid gland, sclerosing epithelioid fibrosarcoma, biphenotypic sinonasal sarcoma, sarcomatoid carcinoma, and metastatic low-grade endometrial sarcoma [6,11,14,17]. Aberrant/irregular PAX8 expression in these tumors may lead to misdiagnosis. Diffuse pan-cytokeratin expression associated with negative STAT6 expression is highly suggestive of an anaplastic carcinoma, rather than solitary fibrous tumors [6,11]. Spindle cell carcinoma should always be ruled out in the submucosal stratum of the head and neck area, while malignant melanoma should be taken into consideration [6,25].

Sinonasal glomangiopericytoma, a differential diagnosis for solitary fibrous tumor of the sinonasal tract, contains bland, monotonous fusiform cells with intervening characteristic staghorn vasculature, lacking hypocellular and hypercellular areas, thick collagen bundles, and perivascular hyalinization [6]. Tumor cells express diffuse positive expression for smooth muscle actin and β-catenin, while negative reaction for CD34 and STAT6 [6].

Biphenotypic sinonasal sarcoma is formed of bland fusiform cells with a herringbone pattern, thin collagen fibers, and staghorn vasculature. Tumor cells are generally positive for smooth muscle actin and S100 protein stains [6]. A weak reaction for CD34, desmin, MyoD1, and myogenin is observed, while SOX10 expression is negative [6,14,25]. Cytoplasmic expression for STAT6 may be present, but nuclear expression is not [6,16]. The typical molecular alteration in biphenotypic sinonasal sarcoma is PAX3-MAML3 gene fusion [6,14].

Nasopharyngeal angiofibroma contains bland stromal cells and copious vasculature. Tumor cells are positive for androgen receptors, actin, smooth muscle, and β-catenin protein, while negative for STAT6 stains [6,14,16].

Poorly differentiated sinonasal sarcoma is composed of fusiform or epithelioid cells, with a storiform or sheet-like pattern, intervening staghorn vasculature, and collagen bundles [6]. Positive expression for CD99 and Bcl2 is present in both sinonasal sarcoma and solitary fibrous tumors. Rarely, some cases with diffuse positive expression for TLE1 and STAT6 expression were found, yet always with negative CD34 reaction [6]. For clarifying the diagnosis of the challenging cases, the molecular signature of the sinonasal sarcoma that is translocation t(X;18) can be used [6,25].

Malignant peripheral nerve sheath tumors contain alternating hypocellular and hypercellular areas, focal HPC-like dispositions, CD34-positive reaction, and focal nerve sheath morphology. Loss of H3K27me expression, alongside some positive expressions of STAT6, S100, SOX10, and GFAP, is present as well [6].

Benign tumors of the peripheral nerve sheet, namely schwannoma and neurofibroma, could be used in the differential diagnosis of solitary fibrous tumors. Alternating hypocellular and hypercellular areas alongside hyalinized blood vessels are seen in benign schwannomas, although wavy nuclei with tapered ends could give away the diagnosis [6,36]. Both neurofibroma and schwannoma are positive for SOX10 and S100 protein, negative for STAT6; meanwhile, neurofibroma is also positive for CD34 [6,25].

The benign giant-cell angiofibroma contains spindle cells, bland fibrous tissue stroma, and floret-type giant cells, and remarkable immunoreactivity with CD34 [9].

This “chameleonic” capacity of the solitary fibrous tumor is reflected in our understanding of the contemporary spectrum of tumors, particularly in the head and neck region, helping to better define their biological potential and clinical behavior [11].

Although generally benign tumors, malignant variants of the solitary fibrous tumors have been recognized [2,7,9]. Malignant forms are usually larger in size (>50 mm), hypercellular with moderate to increased cytological atypia, infiltrative margins, focal necrosis, and a marked mitotic rate (>4 mitoses/10 high-power microscopic fields) [7,8,9,14,17,37]. According to Gold et al., the presence of a malignant feature was associated with both worse local recurrence-free survival and metastasis-free survival of the patients, factors that led to poor prognosis [8,38]. Around 5–20% of pleural solitary fibrous tumors may have malignant behavior, while just about 7% of the head and neck solitary fibrous tumors, according to Cox et al., have atypical or malignant features [9]. Chung et al. mentioned that not all cases with malignant aspects behave aggressively [7]. Gholami et al. discovered that the histopathological classification of malignant transformation has no association with an increased risk of recurrence or even death [7,39]. In our research, no malignant solitary fibrous tumors were noted.

The conventional notion of benign and malignant solitary fibrous tumors has evolved over the years, due to the ability to mimic various clinical aspects; therefore, some risk-assessment models have been suggested [6]. The criteria for malignancy have been inconsistent throughout published studies [40]. Demicco et al. presented a model that is the most widely practiced one, which predicts the risk of metastasis and tumor-related death, dividing the tumors into low, intermediate, and high-risk categories, based on some parameters such as: patient’s age, size of the tumor, mitotic count (mitoses/mm^2^), presence of hemorrhage/necrosis areas and the presence of sharply demarcated anaplastic/poorly differentiated foci [1,6,16,37,38,40].

Complete local surgical excision is the primary treatment option [6,8,9,12,41]. Gold et al. reported that the presence of positive surgical margins, macroscopically or microscopically, was associated with a poorer outcome, regarding local recurrence and metastasis [8,38]. Cox et al. revealed that 4 out of 9 cases with positive margins presented recurrence, while only 1 case with negative margins among the other 10 presented the same [2,9]. Taking into consideration the degree of difficulty or even the possibility of resection, due to the nature of the head and neck region, successful surgical treatment remains associated with a better prognosis [2,8]. We would like to mention that any of the classifications mentioned in the literature cannot be applicable for orbital solitary fibrous tumors, ascribed to difficulty in obtaining complete excision in this particular site, which increases the risk of obtaining low or no surgical margins and even local invasion [12].

Adjuvant therapies involve preoperative tumor embolization therapy and postoperative chemotherapy or radiation therapy opted for in patients with aggressive histological aspects, positive surgical margins, local invasion, or recurrence [2,6,8,14,15,42]. There are no suggested standardized chemotherapeutic protocols for these tumors [17].

Postoperative monitoring is important because the tumor can recur even after several years [2,12,42].

In the head and neck area, loco-regional recurrence rates can reach 40%, while distant metastasis rates are 16.7% [4,6,11]. In contrast, Cox et al. mentioned that after complete excision, recurrence is rare, appearing only in incompletely excised cases [9]. Naturally, due to its rarity, findings about the clinical behavior and, inclusively, treatment variations of head and neck solitary fibrous tumors are restricted [6,11,15]. Smith et al. found that both size and mitotic index remain primary prognostic factors for recurrence. Moreover, they observed that 67% of histologically positive margins did not correlate with further local recurrence [11]. Demicco et al. reported local recurrence in 3 of 12 cases and metastasis in 2 of 12 cases [1,6,11]. The importance of complete local excision of the tumors is of great value, especially knowing that an incomplete resection with positive surgical margins may lead to recurrence [9]. Less frequent recurrence has been observed in tumors of the oral cavity [6]. The data obtained from our research relates 100% recurrence for the tumors with positive margins, while one case reported negative margins with associated recurrence. For one of the cases, it was not possible to provide the status of the margins; however, the recurrence was present.

Some studies described that the presence and overexpression of a telomerase reverse transcriptase promoter mutation (TERT) could be associated with a decreased disease-free survival [6,7].

In previous studies, Smith et al. published a median time to recurrence being 19 months, while Baněčková et al. reported 36 months [7,11,14]. Chung et al. mentioned a median time to recurrence of 28.5 months, while the latest time was 113 months, the last one being found in the literature as late as 228 months, nearly 20 years from initial treatment [7,11]. That being said, the necessity of long-term clinical and imaging follow-up should be reiterated [7,17].

Smith et al. concluded that Demicco et al.’s model is of limited utility in the head and neck region because of the higher recurrence rate (~40%) and lower metastatic rate (~6%), in contrast to the lower recurrent rate (10%) and higher metastatic rate (26%) in other sites [6,11]. They suggested an additive joint prognostic model conferring tumor size (>5 cm) and mitotic count (>4/10 HPF) to be associated with the risk of recurrence [6,15]. Thompson et al. found an accurate risk stratification model of the orbital tumors based on patient age (>45 years), size of the tumor (>3 cm), number of mitoses (>4 mitoses/2 mm^2)^, necrosis, moderate to high cellularity, and pleomorphism as well [6,24].

Risk stratification was performed using six scoring systems. The majority of the cases were categorized as low risk across all systems, ranging from 58% (Sugita system) to 96% (Salas system). The intermediate-risk assignments varied more widely, with the Sugita score classifying 38%, compared to only 4% in the Salas system, while high-risk designations were rare across all scoring models. Thus, 4% of the cohort was classified as high risk in the Sugita, Huang, and Thompson scores, while 12% in the Demicco system. G-score and Salas score did not identify any high-risk patients. These discrepancies reflect the heterogeneity of risk-assessment criteria among the scoring systems, although overall trends remained broadly consistent.

The presence of the metastases has been noted in sites other than the head and neck region; for the head and neck, solitary fibrous tumors are infrequent, with only a few mentioned in the written reports [7,9,11]. In the largest meta-analysis of head and neck cases, numbering 587, Stanisce et al. mentioned 2 cases with metastatic features [7,15]. The pulmonary site was the most common one regarding distant metastasis in all the literature [7,43]. No metastases of the solitary fibrous tumor were found in the cases presented in this study.

Death caused by this disease can occur in up to 8% of cases [11]. In the group of patients mentioned in this study, the death of one patient was mentioned, the cause being cardiorespiratory decompensation—a medical condition unrelated to the solitary fibrous tumor in question [6].

## 5. Conclusions

This manuscript is a retrospective study of all the solitary fibrous tumors diagnosed in the head and neck region in the western part of Romania, since 2017. The task of establishing the diagnosis is nonetheless a challenging one, knowing the chameleonic ability of these tumors, on top of which the rarity in the head and neck region can be added. The final diagnosis relies solely on histopathological examination using immunohistochemical reactions. The therapeutic gold standard is complete local surgical excision, respecting and preserving the vital adjacent structures, which hinders the intervention. Periodic clinic and imagistic long-term follow-up is of major importance regarding the monitoring of the disease with its characteristic recurrence, malignancy transformation, and even possible metastasis. This research describes our experience with head and neck solitary fibrous tumors, the therapeutic decisions, and the morphopathological features, while reviewing the published cases in the specialized English literature. The clinical data of the solitary fibrous tumors are nearly entirely collected from published case reports and series; therefore, we consider the importance of researching and reporting all diagnosed cases to be no less than meaningful, so the awareness of this versatile tumor could be brought to light, including in the differential diagnosis of head and neck pathogenesis.

## Figures and Tables

**Table 1:** Data related to the antibodies used for the immunohistochemical study.

Antibody	Substrate	Clone	Dilution
^1^ STAT6	Monoclonal, rabbit	YE361	1:50
^2^ CD34	Monoclonal, mouse	QBEnd/10	RTU
^3^ CD99	Monoclonal, mouse	PCB1	1:50
^4^ EMA	Monoclonal, mouse	GP1.4	1:300
^5^ Bcl2	Monoclonal, rabbit	SP66	1:100
^6^ SMA	Monoclonal, mouse	Asn-1	1:50
^7^ S100	Polyclonal, rabbit	EP32	1:100
^8^ DES	Monoclonal, mouse	DE-R-11	1:75
^9^ Ki-67	Monoclonal, mouse	MM1	1:200
^10^ CD3	Monoclonal, mouse	LN10	1:500
^11^ CD4	Monoclonal, mouse	4B12	1:100
^12^ CD8	Monoclonal, mouse	4B11	1:500
^13^ CD5	Monoclonal, mouse	4C7	1:100
^14^ CD20	Monoclonal, mouse	L26	RTU
^15^ CD1a	Monoclonal, mouse	MTB1	RTU
^16^ CD117	Monoclonal, rabbit	EP10	1:200

^1^ STAT6: Signal Transducer and Activator of Transcription 6; ^2^ CD34: Cluster of Differentiation 34; ^3^ CD99: Cluster of Differentiation 99; ^4^ EMA: Epithelial membrane antigen; ^5^ Bcl2: B-cell lymphoma 2; ^6^ SMA: Smooth Muscle Actin; ^7^ S100: S100 Protein; ^8^ DES: Desmin; ^9^ Ki-67: Marker of Proliferation Ki-67; ^10^ CD3: Cluster of Differentiation 3; ^11^ CD4: Cluster of Differentiation 4; ^12^ CD8: Cluster of Differentiation 8; ^13^ CD5: Cluster of Differentiation 5; ^14^ CD20: Cluster of Differentiation 20; ^15^ CD1a: Cluster of Differentiation 1a; ^16^ CD117: KIT proto-oncogene receptor tyrosine kinase.

**Table 2:** Clinical, epidemiological, and data revealing the treatment and outcome of the analyzed tumors.

Variable	All Patients (*N* = 26)
Sex (male)	15 (57.7%)
Age (years)	55 (37.5–64)
Site ^(a)^	
Auricular and periauricular	7 (26.92%)
Nasal region	6 (23.08%)
Orbital and periorbital	5 (19.23%)
Labial region	2 (7.69%)
Other regions *	6 (23.08%)
Size ^(b)^	1.3 (0.7–2.7)
Surgical excision ^(a)^	21 (80.77%)
Incisional biopsy ^(a)^	5 (19.23%)
^1^ RTx ^(a)^	1 (3.85%)
^2^ CTX ^(a)^	4 (15.38%)
Recurrence ^(a)^	6 (23.08%)
Time to recurrence (months) ^(b)^	17 (6.50–77)
Recurrence size ^(b)^	2.65 (2.05–3.40)

* Other regions include one case in each of nasopharyngeal, oropharyngeal, latero-cervical, maxillary, and jugal region; ^(a)^ Absolute count (percentage); ^(b)^ Median of tumor diameter in cm (^3^ IQR); ^1^ RTx: Radiotherapy; ^2^ CTX: Chemotherapy; ^3^ IQR: interquartile range.

**Table 3:** Histopathological characteristics in Hematoxylin–Eosin staining of the tumors—absolute count (percentage).

Pathological Features	All Patients
Spindle cells	19 (73.08%)
Spindle and epitheloid cells	7 (26.92%)
Low cell density	14 (53.84%)
Moderate cell density	6 (23.08%)
High cell density	6 (23.08%)
≤4 mitoses/10 high-power field	21 (80.77%)
>5 mitoses/10 high-power field	5 (19.23%)
Low pleomorphism	17 (65.39%)
Moderate pleomorphism	4 (15.38%)
High pleomorphism	5 (19.23%)
Focal areas of necrosis	3 (11.54%)

**Table 4:** Immunohistochemical profile of solitary fibrous tumors of the head and neck: diagnostic and prognostic immunohistochemical markers.

Case	^1^ STAT6	^2^ CD34	^3^ EMA	^4^ Bcl2	^5^ SMA	^6^ S100	^7^ DES	^8^ CD99	^9^ Ki-67
1	^10^ +++, Diffuse, 100%	+++, Diffuse, 90%	^11^ -	^12^ ++, Diffuse, 98%	-	-	-	+++, Diffuse, 80%	15%
2	+++, Diffuse, 100%	^13^ +, Diffuse, 100%	-	+, Diffuse, 98%	-	-	-	+++, Diffuse, 90%	10%
3	+++, Diffuse, 100%	+++, Diffuse, 100%	-	+, Diffuse, 100%	-	-	-	+++, Diffuse, 90%	30%
4	+++, Diffuse, 100%	+++, Diffuse, 100%	-	++/+++, Diffuse, 98%	-	-	-	+++, Diffuse, 100%	5%
5	++/+++, Diffuse, 90%	+++, Diffuse, 80%	++, Focal, 30/%	+++, Diffuse, 98%	-	-	-	-	3%
6	+++, Diffuse, 100%	+++, Diffuse, 80%	+++, Focal, 50%	++, Focal, 60%	-	-	-	+++, Diffuse, 100%	15%
7	+++, Diffuse, 100%	+++, Focal, 50%	-	++/+++, Diffuse, 90%	+, focal, 10%	-	-	+++, Diffuse, 90%	10%
8	+++, Diffuse, 100%	+++, Diffuse, 98%	-	+++, Diffuse, 98%	-	-	-	+++, Diffuse, 70%	5%
9	+++, Diffuse, 100%	+++, Diffuse, 100%	-	+++, Diffuse, 100%	-	-	-	+++, Diffuse, 70%	5%
10	++, Diffuse, 100%	+++, Diffuse, 100%	-	+++, Diffuse, 100%	-	-	-	-	3%
11	+++, Diffuse, 100%	+/++, Diffuse, 95%	-	+++, Diffuse, 100%	-	-	-	-	15%
12	+++, Diffuse, 100%	+, Diffuse, 100%	-	+++, Diffuse, 100%	-	-	-	+++, Diffuse, 90%	20%
13	+++, Diffuse, 100%	+++, Diffuse, 100%	+	+++, Diffuse, 100%	-	-	-	-	7%
14	+++, Diffuse, 100%	+++, Diffuse, 100%	+	++, Diffuse, 90%	-	-	-	+++, Diffuse, 50%	25%
15	+++, Diffuse, 100%	-	-	+, Diffuse, 95%	-	-	-	+++, Diffuse, 100%	5%
16	+++, Diffuse, 100%	+++, Diffuse, 100%	-	+, Diffuse, 85%	-	-	-	+++, Diffuse, 90%	5%
17	+++, Diffuse, 100%	+++, Diffuse, 100%	-	+++, Diffuse, 100%	-	-	-	+++, Diffuse, 90%	15%
18	+++, Diffuse, 100%	+++, Diffuse, 100%	-	+++, Diffuse, 100%	-	-	-	+++, Diffuse, 90%	7%
19	+++, Diffuse, 100%	+++, Diffuse, 100%	-	+++, Diffuse, 80%	-	-	-	+++, Diffuse, 95%	5%
20	+++, Diffuse, 95%	+++, Diffuse, 100%	-	+++, Diffuse, 100%	-	-	-	-	5%
21	+++, Diffuse, 100%	+++, Diffuse, 100%	-	+++, Diffuse, 100%	-	-	-	+++, Diffuse, 90%	5%
22	+++, Diffuse, 100%	+++, Diffuse, 100%	-	+++, Diffuse, 100%	-	-	-	+++, Diffuse, 75%	15%
23	+++, Diffuse, 100%	-	-	+++, Diffuse, 100%	-	-	-	+++, Diffuse, 90%	10%
24	+++, Diffuse, 100%	+++, Diffuse, 100%	-	+++, Diffuse, 100%	-	-	-	+++, Diffuse, 80%	20%
25	+++, Diffuse, 90%	+++, Diffuse, 100%	-	+++, Diffuse, 100%	-	-	-	+++, Diffuse, 100%	10%
26	+++, Diffuse, 100%	+++, Diffuse, 100%	-	+++, Diffuse, 100%	-	-	-	+++, Diffuse, 90%	20%

^1^ STAT6: Signal Transducer and Activator of Transcription 6; ^2^ CD34: Cluster of Differentiation 34; ^3^ EMA: Epithelial membrane antigen; ^4^ Bcl2: B-cell lymphoma 2; ^5^ SMA: Smooth Muscle Actin; ^6^ S100: S100 Protein; ^7^ DES: Desmin; ^8^ CD99: Cluster of Differentiation 99; ^9^ Ki-67: Marker of Proliferation Ki-67; ^10^ +++: Intense positive reaction; ^11^ -: Negative reaction; ^12^ ++: Moderate positive reaction; ^13^ +: Low positive reaction.

**Table 5:** The IHC evaluation of the tumor immune microenvironment—scoring system ranging.

Marker	Score 0	Score 1	Score 2	Score 3
^1^ CD20	13 (50%)	4 (15%)	2 (8%)	7 (27%)
^2^ CD3	0 (0%)	1 (4%)	4 (15%)	21 (81%)
^3^ CD4	1 (4%)	21 (81%)	4 (15%)	0 (0%)
^4^ CD5	0 (0%)	0 (0%)	5 (19%)	21 (81%)
^5^ CD8	2 (8%)	17 (65%)	7 (27%)	0 (0%)
^6^ CD1a	22 (85%)	4 (15%)	0 (0%)	0 (0%)
^7^ CD117	23 (88%)	3 (12%)	0 (0%)	0 (0%)

^1^ CD20: Cluster of Differentiation 20; ^2^ CD3: Cluster of Differentiation 3; ^3^ CD4: Cluster of Differentiation 4; ^4^ CD5: Cluster of Differentiation 5; ^5^ CD8: Cluster of Differentiation 8; ^6^ CD1a: Cluster of Differentiation 1a; ^7^ CD117: KIT proto-oncogene receptor tyrosine kinase.

**Table 6:** Comparative classification of risk according to six established prognostic scores.

Score	Low Risk	Intermediate Risk	High Risk
Demicco	17 (65%)	6 (23%)	3 (12%)
Sugita	15 (58%)	10 (38%)	1 (4%)
G-score	19 (73%)	7 (27%)	0 (0%)
Huang	23 (88%)	2 (8%)	1 (4%)
Salas	25 (96%)	1 (4%)	0 (0%)
Thompson	20 (77%)	5 (19%)	1 (4%)

**Table 7:** Comparative performance of each score to predict the recurrence of the tumor.

Score	AUC (95% CI)	Sensitivity	Specificity
Demicco	0.771 (0.518–0.999)	50%	100%
Sugita	0.896 (0.805–0.987)	100%	75%
G-score	0.758 (0.537–0.980)	66.7%	85%
Huang	0.750 (0.531–0.969)	50%	100%
Salas	0.583 (0.420–0.747)	16.7%	100%
Thompson	0.896 (0.726–0.999)	83.3%	95%

## Data Availability

The data that support the funding for this study are available from the corresponding author upon reasonable request.

## Supplementary Materials

The following supporting information can be downloaded at: https://www.mdpi.com/article/10.3390/diagnostics15212721/s1, Table S1: Clinical and epidemiological data; Table S2: Data revealing the treatment and outcome; Table S3: Morphological aspects in Hematoxylin-Eosin staining.
