# Supplementary material for: The Solitary Fibrous Tumor, the Chameleon of the Head and Neck Region—Clinical, Epidemiological, and Histopathological Aspects
Source: Diagnostics (Basel). 2025 Oct 27;15(21):2721. doi: 10.3390/diagnostics15212721 (PMC12608949; doi:10.3390/diagnostics15212721)
Supplement: Supplementary file 1 [file diagnostics-15-02721-s001.zip › diagnostics-3910197-supplementary.pdf]

The Solitary Fibrous Tumor, the Chameleon of the Head and Neck Region—Clinical, Epidemiological and Histopathological Aspects

Supplemental Table S1. Clinical and epidemiological data.

Case	Age	Gender	Site	Signs and symptoms	Size
1	57	[1] M	Right supraorbital region	Physiognomic disturbance	4.2 cm
2	19	M	Nasopharynx	Dyspnea, dysphonia	2 cm
3	25	M	Right nasal septum	Right nasal obstruction	2.4 cm
4	53	[2] F	Outer corner of the left eye	Physiognomic disturbance	0.5 cm
5	39	F	Left helix	Painless	0.5 cm
6	34	M	Palpebral	Painless	0.2 cm
7	42	M	Inner corner of the left eye	Slight discomfort	1.3 cm
8	69	M	Right preauricular region	Painless	1 cm
9	37	M	Nasolabial	Painless	0.6 cm
10	24	M	Nasal fosa	Painless	0.7 cm
11	43	M	Left genian region	Painless	1.3 cm
12	54	F	Right auricular region	Painless	0.5 cm
13	35	M	Right nasal cavity	Right nasal obstruction, rhinorrhea, dysphonia	0.7 cm
14	32	M	Upper labial mucosa	Slight discomfort	0.5 cm
15	81	F	Upper labial region	Slight discomfort	0.7 cm
16	66	F	Right auricular region	Physiognomic disturbance	2.5 cm
17	56	F	Left tympanic region	Unilateral hearing loss	0.7 cm
18	69	F	Right inferior nasal turbinate	Right nasal obstruction	1 cm
19	56	M	Right laterocervical region	Painless	1.5 cm
20	59	M	Right retroauricular region	Painless	1.5 cm
21	40	F	Right temporo-zygomatic fossa	Painless	2.7 cm
22	64	F	Left maxillary vestibule	Discomfort in mastication	11 cm
23	65	M	Left middle meatus, extension to the posterior wall of the nasopharynx	Dysphagia, odynophagia, bilateral nasal obstruction, oral breathing	6 cm
24	58	M	Left jugal region	Painless	5.5 cm
25	74	F	Left intraorbital region	Mass effect, visual acuity	5.1 cm
26	64	F	Oropharynx	Dysphagia, dysphonia	3 cm

[1] M: Masculine; [2] F: Feminine.

Supplemental Table S2. Data revealing the treatment and outcome.

Case	Surgical treatment	Surgical approach	Postoperative margins	[1] CTX/[2] RTx	Recurrence	Time to recurrence (months)	Recurrence size (cm)	Death
1	Excisional biopsy	Cutaneous	[3] +	No	Yes	20	2	No
2	Excisional biopsy	Endonasal	+	No	Yes	4	2.2	No
3	Excisional biopsy	Endonasal	[4] -	No	No	[5] NA	NA	No
4	Excisional biopsy	Cutaneous	-	No	No	NA	NA	No
5	Excisional biopsy	Cutaneous	-	No	No	NA	NA	No
6	Excisional biopsy	Cutaneous	-	No	No	NA	NA	No
7	Excisional biopsy	Cutaneous	-	No	No	NA	NA	No
8	Excisional biopsy	Cutaneous	-	No	No	NA	NA	No
9	Excisional biopsy	Cutaneous	-	No	No	NA	NA	No
10	Excisional biopsy	Endonasal	-	No	No	NA	NA	No
11	Excisional biopsy	Intraoral	-	No	No	NA	NA	No
12	Excisional biopsy	Cutaneous	-	No	No	NA	NA	No
13	Excisional biopsy	Endonasal	-	RTx	No	NA	NA	No
14	Excisional biopsy	Intraoral	-	No	No	NA	NA	No
15	Excisional biopsy	Cutaneous	-	No	No	NA	NA	No
16	Excisional biopsy	Cutaneous	-	No	No	NA	NA	No
17	Incisional biopsy	Cutaneous	NA	RTx	No	NA	NA	No
18	Incisional biopsy	Endonasal	NA	No	No	NA	NA	No
19	Excisional biopsy	Cutaneous	-	No	No	NA	NA	No
20	Excisional biopsy	Cutaneous	-	No	No	NA	NA	No
21	Excisional biopsy	Cutaneous	-	No	No	NA	NA	No
22	Excisional biopsy	Intraoral	-	RTx	Yes	168	1.4	No
23	Excisional biopsy	Intraoral	-	No	No	NA	NA	No
24	Incisional biopsy	Intraoral	+	RTx (after [6] R3)	Yes	14/28/34/42	3/5.2/4.1/3.5	No
25	Incisional biopsy	Cutaneous	NA	CTX	Yes	96	3.5	No
26	Incisional biopsy	Intraoral	NA	No	No	3	3.1	Yes (after 4 months)

[1] CTX: Chemotherapy; [2] RTx: Radiotherapy; [3] +: Positive; [4] -: Negative [5] NA: not applicable; [6] R3: the third recurrence.

Supplemental Table S3. Morphological aspects in Hematoxylin-Eosin staining.

Case	Dominant constituent cell	Histopathological subtype	Cell density	Mitotic index	Cellular atypia	Necrosis
1	Spindle	Cellular	High	4	Minim	No
2	Spindle, epiteliod	Mixoid	Low	5	High	No
3	Spindle	Classic	Moderate	2	Minim	No
4	Spindle	Classic	Low	1	Minim	No
5	Spindle	Classic	Low	1	Minim	No
6	Spindle	Classic	Moderate	4	Minim	No
7	Spindle	Classic	Low	3	Moderate	No
8	Spindle	Classic	Moderate	2	Minim	No
9	Spindle	Classic	Moderate	1	Minim	No
10	Spindle	Classic	Low	1	Minim	No
11	Spindle, epiteliod	Cellular	High	4	Minim	No
12	Spindle	Classic	Low	4	High	No
13	Spindle	Classic	Low	2	Minim	No
14	Spindle	Classic	Low	5	Minim	No
15	Spindle	Classic	Low	2	Minim	No
16	Spindle	Paucicellular sclerotic collagen	Low	1	Moderate	No
17	Spindle	Cellular	High	3	Minim	No
18	Spindle	Mixoid	Moderate	2	Minim	No
19	Spindle	Mixoid	Moderate	2	Moderate	No
20	Spindle	Mixoid	Low	1	Moderate	No
21	Spindle	Classic	Low	2	Minim	No
22	Spindle, epiteliod	Cellular	High	7	High	Focal areas
23	Spindle, epiteliod	Cellular	High	3	Minim	No
24	Spindle, epiteliod	Mixoid	High	5	High	Focal areas
25	Spindle, epiteliod	Mixoid	Low	8	High	Focal areas
26	Spindle, epiteliod	Classic	Low	4	Minim	No
